# Diffusion Kurtosis Imaging as a Prognostic Marker in Osteosarcoma Patients with Preoperative Chemotherapy

**DOI:** 10.1155/2020/3268138

**Published:** 2020-09-26

**Authors:** Chenglei Liu, Yue Xing, Dongmin Wei, Qiong Jiao, Qingcheng Yang, Dapeng Lei, Xiaofeng Tao, Weiwu Yao

**Affiliations:** ^1^Department of Radiology, Shanghai Ninth People's Hospital, Shanghai Jiao Tong University School of Medicine, Shanghai, China; ^2^Department of Radiology, Shanghai Jiao Tong University Affiliated Sixth People's Hospital, Shanghai, China; ^3^Department of Otorhinolaryngology, Qilu Hospital, Shandong University, Shandong 250012, China; ^4^Key Laboratory of Otolaryngology, NHFPC (Shandong University), Shandong 250012, China; ^5^Department of Pathology, Shanghai Jiao Tong University Affiliated Sixth People's Hospital, Shanghai, China; ^6^Department of Orthopaedics, Shanghai Jiao Tong University Affiliated Sixth People's Hospital, Shanghai, China

## Abstract

**Background:**

The accurate prediction of prognosis is key to prompt therapy adjustment. The purpose of our study was to investigate the efficacy of diffusion kurtosis imaging (DKI) in predicting progression-free survival (PFS) and overall survival (OS) in osteosarcoma patients with preoperative chemotherapy.

**Methods:**

Thirty patients who underwent DKI before and after chemotherapy, followed by tumor resection, were retrospectively enrolled. The patients were grouped into good responders (GRs) and poor responders (PRs). The Kaplan-Meier and log-rank test were used for survival analysis. The association between the DKI parameters and OS and PFS was performed by univariate and multivariate Cox proportional hazards models.

**Results:**

Significantly worse OS and PFS were associated with a lower mean diffusivity (MD) after chemotherapy (HR, 5.8; 95% CI, 1.5-23.1; *P* = 0.012 and HR, 3.5; 95% CI, 1.2-10.1: *P* = 0.028, respectively) and a higher mean kurtosis (MK) after chemotherapy (HR, 0.3; 95% CI, 0.1-0.9; *P* = 0.041 and HR, 0.3; 95% CI, 0.1-0.8; *P* = 0.049, respectively). Likewise, shorter OS and PFS were also significantly associated with a change rate in MD (CR MD) of less than 13.53% (HR, 8.6; 95% CI, 1.8-41.8; *P* = 0.007 and HR, 2.9; 95% CI, 1.0-8.2; *P* = 0.045, respectively). Compared to GRs, PRs had an approximately 9- and 4-fold increased risk of death (HR, 9.4; 95% CI, 1.2-75; *P* = 0.034) and progression (HR, 4.2; 95% CI, 1.2-15; *P* = 0.026), respectively.

**Conclusions:**

DKI has a potential to be a prognostic tool in osteosarcoma. Low MK and high MD after chemotherapy or high CR MD indicates favorite outcome, while prospective studies with large sample sizes are warranted.

## 1. Introduction

Osteosarcoma is one of the most common primary malignant bone tumors in children and adolescents, and approximately 75-80% of osteosarcoma involves appendicular bone [1]. The importance of preoperative neoadjuvant chemotherapy in the treatment of osteosarcoma has been confirmed, and effective chemotherapy has dramatically improved patient survival rates, contributing to a 5-year survival rate increase from 20% to 70% [2]. Currently, increasing evidence has shown that chemotherapy-induced tumor necrosis is the strongest known predictive indicator for survival [3, 4]. In clinical practice, the early identification of chemotherapy response is key to prompting treatment regimen adjustments, as ineffective chemotherapy has the potential to increase the risk of complications and mortality or form resistant clones [5]. However, the crucial issue is the lack of imaging features for monitoring chemotherapy response in vivo. Suboptimal histologic response to neoadjuvant chemotherapy can be assessed only from the postoperative specimen after the completion of neoadjuvant chemotherapy. Therefore, there is an urgent need to discover imaging surrogates that are reliable preoperative prognostic indictors.

Currently, several imaging modalities, including computed tomography (CT), magnetic resonance imaging (MRI), and PET/CT, play a crucial role in monitoring the neoadjuvant chemotherapy response of osteosarcoma [6, 7]. Conventional diffusion-weighted imaging (DWI), a type of functional MR imaging, has been used to reflect chemotherapy-induced tumor necrosis. A few studies have demonstrated that apparent diffusion coefficient (ADC) values were significantly associated with tumor necrosis after surgery [8–16]. However, this imaging modality is based on the assumption of a Gaussian distribution of water diffusion in tissue. In reality, water diffusion deviates from this pattern due to the presence of intracellular and extracellular complexes in the tissue microstructure. Thus, DWI may result in an inaccurate reflection of tissue microstructure heterogeneity and complexity.

In contrast to DWI, diffusion kurtosis imaging (DKI) provides a method which might reflect more precisely the diffusional heterogeneity of the tumor by quantifying the non-Gaussian diffusion of water molecules in bone tumors. To date, few studies have evaluated chemotherapeutic response using DKI [17, 18]. Recently, DKI has been shown to have the potential to predict survival outcomes in high-grade glioma patients [19]. However, to the best of our knowledge, there is little information regarding whether DKI has the ability to evaluate prognosis in osteosarcoma patients with preoperative chemotherapy. Therefore, our purpose was to investigate the utilization of DKI in prediction of the survival outcome in high-grade osteosarcoma patients.

## 2. Materials and Methods

### 2.1. Study Subjects

Our retrospective study was approved by the Ethics Committee of Shanghai Sixth Hospital, and written informed consent was obtained from each patient. Thirty-three patients were recruited between March 2016 and December 2016. The patients were eligible for inclusion with the following criteria, if they (1) had pathologically proven osteosarcoma and received preoperative chemotherapy followed by surgery, (2) had complete and interpreted data in the MRI database including routine MRI and DKI before and after chemotherapy, (3) had a time interval between biopsy and first MR imaging of more than 7 days and between preoperative MRI and surgery within 2 weeks, (4) had not underwent other preoperative therapy simultaneously, and (5) had available contact information during follow-up. Three patients, for whom DKI had serious motion artifacts, were excluded. Finally, 30 subjects were enrolled, and the detailed clinical information is summarized in Table 1.

### 2.2. MR Imaging Acquisition

The preoperative MR imaging of all patients was performed using a 3.0T MR scanner (MAGETOM, Verio, Siemens Healthcare, Erlangen, Germany), and an eight-channel body array coil was used for signal reception.

DKI was performed using a single shot echo planar imaging sequence with fat suppression. The scan protocol was as follows: axial plane with six *b* values (0, 500, 1000, 1500, 2000, and 2500 s/mm^2^, respectively); TR = 3400 ms; TE = 72 ms; field of view (FOV) = 380 × 380 mm; matrix = 384 × 384; slice thickness = 5 mm; flip angle = 180°; voxel size = 2.0 × 2.0 × 5 mm^3^; and scan duration = 4 min and 37 s. The diffusion gradient of DKI was encoded in three orthogonal directions.

The following scan protocols for routine and contrast-enhanced sequences were as follows: TR = 4000 ms; TE = 104 ms; FOV = 380 × 380 mm; matrix = 384 × 384; and slice thickness = 5 mm for coronal, respectively; sagittal or transverse fat-saturated T2-weighted sequences; and TR = 600 ms; TE = 20 ms; FOV = 380 × 380 mm; matrix = 384 × 384; and slice thickness = 5 mm for coronal, respectively; sagittal or transverse fat-saturated T1-weighted sequences. Fat-saturated T1-weighted contrast-enhanced MRI was performed after injection of 0.1 mmol/kg Gd-DTPA into the cubital vein. The scan protocol was the same as those described above.

### 2.3. MR Image Interpretation

**Figure 1:**
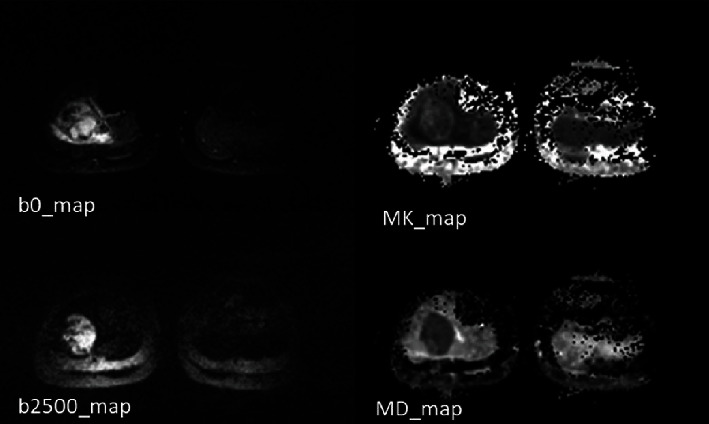
The representative image of diffusion kurtosis imaging (DKI).

Based on the DKI data, diffusivity and kurtosis maps were automatically generated according to the postprocessing software “Body Diffusion Toolbox” (MathWorks, Natick, MA) [20]. The postprocessing procedure for DKI data was previously described in more details [21]. In brief, a Gaussian filter was first used in the software with a full width at half maximum of 3 mm to increase the signal-noise ratio. Then, a voxel-by-voxel fitting of DKI data was performed according to DKI nonlinear equation [22]. The equation is described as follows: *S* = *S*_0_ · exp (−*b* *D* + 1/6 *b*^2^ *D*^2^ *K*), where *S* refers to DWI signal at a particular *b* value, *S*_0_ is the baseline signal without diffusion weighting, *D* is diffusivity, and *K* is kurtosis. Afterwards, two experienced radiologists who were blinded to the tumor necrosis manually drew the regions of interest (ROIs) on the *b*_0_ image and avoided tumor necrotic, cystic, and hemorrhagic areas. Moreover, we used the T2W and enhanced T1W images as a reference [10]. The ROIs were automatically matched on the diffusivity map and kurtosis map. Subsequently, the values of MD and MK for the ROIs were calculated by the software (Figure 1). We defined the change rates in MK and MD before and after neoadjuvant chemotherapy according to the following formulas [14]: CR MK = (pre MK − post MK)/pre MK∗100%and CR MD = (pre MD − post MD)/pre MD∗100%.

### 2.4. Preoperative Chemotherapy and Surgery

Cisplatin (40 mg/m^2^) combined with doxorubicin (25 mg/m^2^) for 3 days was administered by intravenous injection for all patients. Four chemotherapy cycles at intervals of 2-3 weeks were administered. After neoadjuvant chemotherapy, all patients underwent surgery within 3-5 days after follow-up MR imaging.

### 2.5. Pathological Subtype and Response Evaluation

For each patient, the resected specimen was examined by two experienced pathologists, and the tumor necrosis rate was evaluated. Patients with greater than or equal to 90% tumor necrosis were defined as good responders (GRs); otherwise, they were defined as poor responders (PRs) [23].

### 2.6. Follow-Up

Follow-up of all patients was performed by telephone at every 3-month intervals for the first year, at every half-year intervals for years 2-3, and finally at 12-month intervals afterwards. The postoperative evaluation included a clinical examination and radiological analysis (chest CT, localized MRI). We defined progression-free survival (PFS) as the period from the initial DKI date to disease recurrence or the date of the last follow-up. Recurrence included pulmonary metastasis and local recurrence. Overall survival (OS) was defined as the period from the baseline DKI date until death or the date of the last follow-up. The patients who were alive or without recurrence were censored at the latest follow-up. The follow-up ended on December 1^st^, 2019. None of the patients in this study were lost of follow-up.

### 2.7. Statistical Analysis

All statistical analyses were conducted with SPSS 16.0 (SPSS, Inc., Chicago, IL). A *P* value < 0.05 was considered statistically significant. All study subjects were grouped into GRs and PRs for binary comparison. All continuous data fields (pre MK, pre MD, post MK, post MD, CR MK, CR MD) were converted to dichotomous variables using a cut-off point. The receiver operating characteristic (ROC) curve for the prediction of a good histological response (≥90%) was generated to determine the cut-off point that offered the highest sum of sensitivity and specificity of the each variable. The Kaplan-Meier method with the log-rank test was used to compare differences in survival between the groups. The associations between DKI and OS and PFS were evaluated by univariate and multivariate Cox proportional hazards models. To evaluate the joint effect of post MK and MD on survival, the 30 patients were categorized into 3 groups: group 1: patients with post MK < 0.80 and post MD ≥ 1.66 mm^2^/s × 10^−3^ (*n* = 16); group 2: patients with post MK ≥ 0.80 and post MD < 1.66 mm^2^/s × 10^−3^ (*n* = 7); and group 3: patients with other parameters (*n* = 7). Further stratified analyses were performed by tumor response to chemotherapy.

## 3. Results

### 3.1. Patient Characteristics

As shown in Table 1, this study included 21 males and 9 females with an average age of 17.6 years (range 7-34 years). Twenty-eight patients had osteosarcoma in the lower extremities, and 2 patients had osteosarcoma in the upper extremities. Based on the American Joint Committee on Cancer (AJCC) 8th Edition staging system, 4 of the patients were classified as stage IIA, 20 as stage IIB, and 6 as stage III. According to the histopathological results, 27 of the patients were grouped into the osteoblastic subtype, 1 of the patients into the chondroblastic subtype, and 2 of the patients into the small cell subtype. On the basis of Huvos grade, 13 patients had GRs, and the others had PRs. The mean follow-up period was 36 months, ranging from 8 months to 47 months. Five patients experienced local recurrence, and 10 patients experienced lung or other organ metastasis. A total of 10 patients died at the end of study.

### 3.2. DKI-Related Parameters

The DKI-related parameters before and after chemotherapy are summarized in Table 2. For all patients, MK before chemotherapy (pre MK) ranged from 0.58 to 1.46 (mean, 0.92) and MK after chemotherapy (post MK) ranged from 0.46 to 1.14 (mean, 0.78). CR MK ranged from a decrease of 68.8% to an increase of 40.2%. MD before chemotherapy (pre MD) ranged from 0.78 to 1.78 mm^2^/s × 10^−3^ (mean, 1.29 mm^2^/s × 10^−3^) and MD after chemotherapy (post MD) ranged from 0.79 to 2.34 mm^2^/s × 10^−3^ (mean, 1.62 mm^2^/s × 10^−3^). CR MD ranged from a decrease of 43.3% to an increase of 93.5%. The strong reliability and reproducibility of the measurement of DKI-related parameters were previously reported [4]. In brief, the interclass correlation coefficient (ICC) was 0.95 and 0.89 for pre MK and pre MD. For post MD and post MK, the ICC was 0.89 and 0.82.

### 3.3. Association between Histological Response and OS and PFS

The mean OS and PFS were 43.5 and 36.4 months for the 13 GRs and 31.2 and 18.9 months for the 17 PRs. GRs were significantly associated with better OS and PFS (log-rank, *P* = 0.009 and 0.015, respectively). After multivariable analysis with adjustment of other confounders, including age, location, AJCC, and histological subtype and treatment, PRs had an approximately 9- and 4-fold increased risk of overall death (HR, 9.4; 95% CI, 1.2-75; *P* = 0.034) and progression (HR, 4.2; 95% CI, 1.2-15; *P* = 0.026) compared with GRs, respectively.

### 3.4. Association between DKI-Related Parameters and OS and PFS

**Figure 2:**
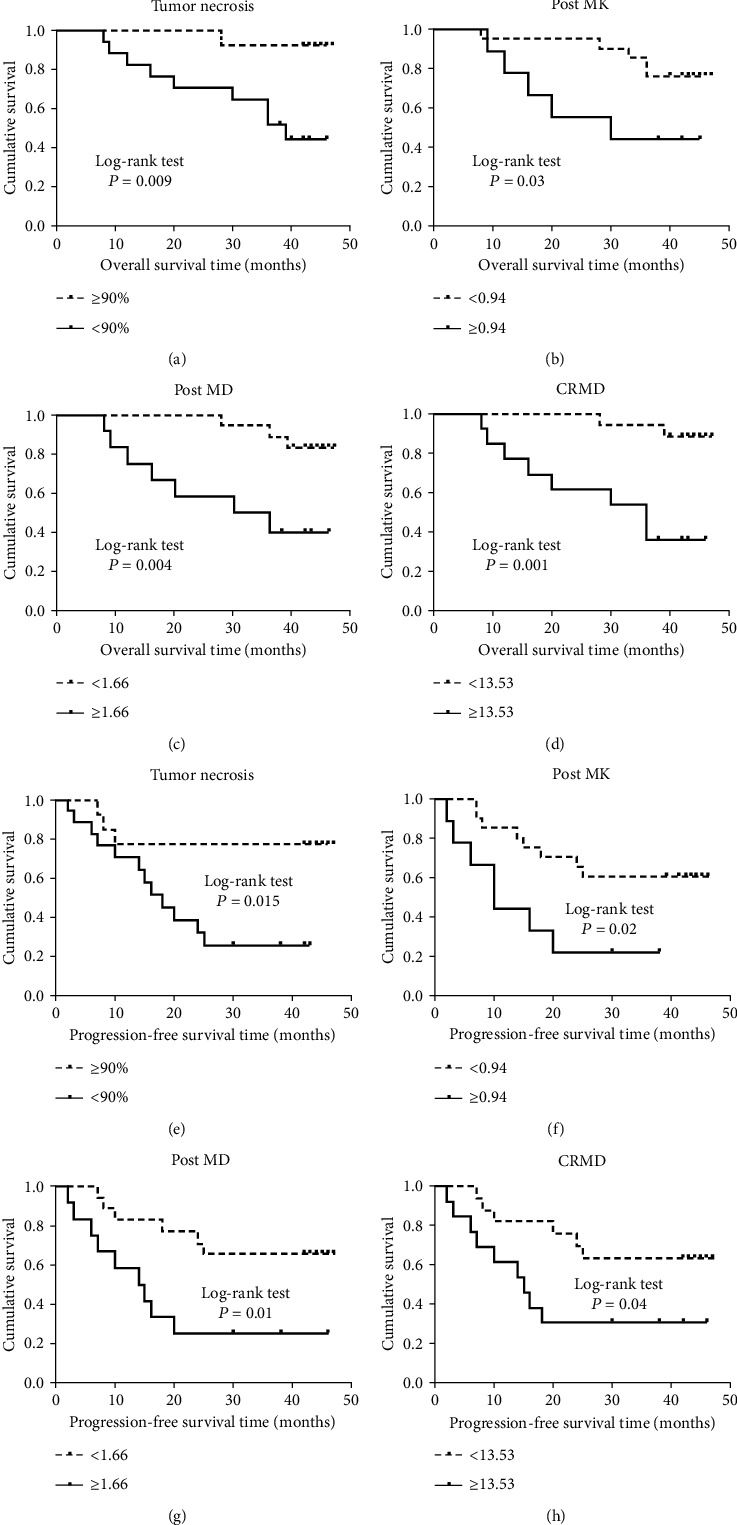
Kaplan-Meier survival curve for overall survival (a–d) and progression-free survival in osteosarcoma patients. Patients with greater than 90% tumor necrosis, high post MD, low post MK, or high CR MD had a significantly better OS and PFS. Post MK: mean kurtosis after neoadjuvant chemotherapy; post MD: mean kurtosis after neoadjuvant chemotherapy; CR MD: change rate in mean kurtosis in mean diffusivity. MD is given in mm^2^/s × 10^−3^.

Our previous study has reported a significant association between tumor necrosis and DKI-related parameters. Therefore, we further explored whether DKI-related parameters were associated with survival. In this study, we found that better OS and PFS were associated with higher values of post MD (log rank, *P* = 0.004 and 0.01, respectively) and with lower values of post MK (log rank, *P* = 0.03 and 0.02, respectively).CR MD greater than or equal to 13.5% was significantly associated with longer OS and PFS (log rank, *P* = 0.001 and 0.04, respectively). Multivariable Cox regression analysis showed that significantly worse OS and PFS were found for patients with a lower post MD (HR, 5.8; 95% CI, 1.5-23.1; *P* = 0.012 and HR, 3.5; 95% CI, 1.2-10.1; *P* = 0.028, respectively) and a higher post MK (HR, 0.3; 95% CI, 0.1-0.9; *P* = 0.041 and HR, 0.3; 95% CI, 0.1-0.8; *P* = 0.049, respectively). Moreover, shorter OS and PFS were also significantly associated with patients with a change of rate less than 13.5% (HR, 8.6; 95% CI, 1.8-41.8; *P* = 0.007 and HR, 2.9; 95% CI, 1.0-8.2; *P* = 0.045, respectively). Unexpectedly, neither pre MK nor pre MD was associated with OS and PFS. Moreover, we did not observe a significant association of CR MK values with the survival (Table 3, Figure 2).

We also evaluated the combined effect of post MK and post MD on survival; we found that the patients with post MK ≥ 0.80 and post MD < 1.66 mm^2^/s × 10^−3^ (group 2) and other parameters (group 3) had approximately 5 and 3 times higher risk of OS compared with the patients with post MK < 0.80 and post MD ≥ 1.66 mm^2^/s × 10^−3^ (group 1), although the association did not reach statistical significance for patients in group 3. Similarly, the same associations were observed for PFS (Table 3).

### 3.5. Association between DKI and OS and PFS Stratified by Histological Response

**Figure 3:**
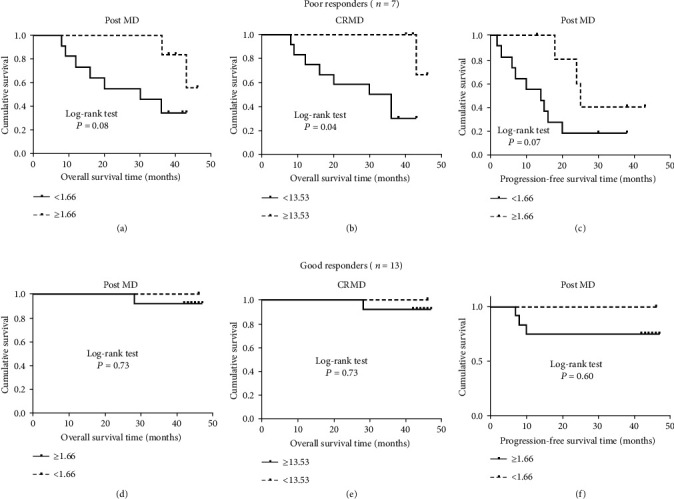
Kaplan-Meier graph of post MK, post MD, and CR MD stratified by tumor necrosis. In poor responders, high post MD was associated with both OS and PFS, whereas lower CR MD was associated with significantly worse OS. In good responders, no obvious differences were found between DKI parameters and OS and PFS. MD is given in mm^2^/s × 10^−3^.

Because of the differences in response to chemotherapy in our patient cohort, we divided our patients into two groups based on tumor necrosis: PRs *vs*. GRs. As shown in Table 4 and Figure 3, for patients with PRs, the log-rank test showed that patients with a lower CR MD had a significant poor OS (*P* = 0.04) than the patients with higher CR MD, and a borderline significant difference in OS and PFS was found for post MD (*P* = 0.080 and 0.070, respectively). After multivariable analysis with adjustment for other confounders, we found that the patients with a lower CR MD had an approximately 6.8 times higher risk of overall death (HR, 6.8; 95% CI, 0.8-58.0; *P* = 0.074) than those with a higher CR MD. Moreover, the patients with higher post MD had an approximately 4 and 3 times lower risk of overall death and progression (HR, 3.7; 95% CI, 0.7-18.7; *P* = 0.077 for OS and HR, 3.1; 95% CI, 0.8-11.7; *P* = 0.069 for PFS), although such associations did not reach a level of statistical significance.

However, there were any such significant associations were found between DKI and OS and PFS among the patients with GRs (Table 4, Figure 3).

## 4. Discussion

In the present study, our findings suggested that post MD, post MK, and CR MD were associated with PFS and OS, which suggested that DKI has potential as a prognostic tool in patients with osteosarcoma. Patients with lower post MK and higher post MD or higher CR MD have an improved outcome.

Histological response induced by neoadjuvant chemotherapy is considered the most reliable prognostic factor for the survival of patients with osteosarcoma [7]. Until now, few studies have investigated the usefulness of diffusion-weighted imaging (DWI) in monitoring the tumor response to chemotherapy in osteosarcoma patients. Thus, there was still no agreement regarding the evaluation of tumor necrosis using DWI-related parameters. Several previous studies showed increased ADC values in response to chemotherapy, and the average ADC change rate before and after chemotherapy could distinguish tumor necrosis based on small sample sizes [14, 24]. However, Oka et al. and Bajpai et al. suggested that the ADC value and change rate in ADC were not associated with tumor necrosis [12, 13]. The wide range of ADC values might be attributed to the heterogeneity of tumor tissue-induced chemotherapy.

Osteosarcoma, especially after neoadjuvant chemotherapy, is characterized by a complex microstructure and heterogeneity. Chemotherapy-induced mitochondrial or organelle swelling, increase in the nuclear-cytoplasmic ratio, or formation of new colonies results in compartmentalization and restricts the free displacement of water molecules, which is attributed to the non-Gaussian diffusion of water molecules [25]. In contrast to DWI, DKI has the potential to illustrate non-Gaussian water diffusion behavior and more accurately reflect and quantify tumor microenvironment complexity [26]. The DKI-related MK and MD are defined as the mean kurtosis and the average diffusivity of all diffusion gradient directions, respectively. Our results suggested that post MD, post MK, and CR MD were predictors of PFS and OS.

Unfortunately, DKI-related parameters before chemotherapy were not associated with PFS or OS, which is in contrast to the results of Wang et al., who suggested that preoperative MK was significantly associated with clinical outcome in patients with high-grade gliomas [19]. This result may be attributed to the intrinsic nature of osteosarcoma. Osteosarcoma may exhibit a faster signal decay than other tissues when *b* factors increase [27]. Moreover, for reducing patient's discomfort and imaging motion artifact, we applied the body DKI model, which required acquisition of only 3 DWIs along the main (*x*, *y*, *z*) orthogonal directions. Although this body DKI model has been used in various types of cancer, it could not reflect the full kurtosis tensor in the tissue, which may affect our result. Thus, further verification was needed in the advanced DKI model. In additional, in this single-center study, although an identical scanner, imaging protocol, and receiver coil were used for a given patient, we did not carry out the correction for nonlinearity of diffusion gradient for DKI, which may potentially affect accuracy of DKI-related quantitative measurement, especially in longitudinal study [28, 29]. In the future, we will employ the DWI quality assurance protocol that Fedeli et al. recommended to further improve DKI accuracy and standardization [30].

Likewise, in contrast to CR MD, CR MK was not significantly associated with OS or PFS. The possible reasons were as follows. On the one hand, this finding may be attributed to the limited sample size. On the other hand, the MD value was decreased, whereas the MK value was not increased in some tumor viscous areas. Furthermore, the area of tumor necrosis with extensive interstitial fibrosis, inflammatory infiltration, and granuloma formation was found in patients who were good responders, which increased the heterogeneity of residual tumors. Last but not least, in this study, we estimated DKI-derived index by fitting DKI model to trace-weighted (TW) images. Although this empirical and straightforward method has been employed in several previous extracranial DKI studies, it could introduce bias and error in the estimation of DKI-derived indices. Accordingly, Giannelli et al. [31] have shown that, for kurtosis values of about 1 (as typically observed in human tissue) and low diffusion anisotropy (<0.2), the absolute percentage error in *K* can range up to 35% or more. Recently, Marzi et al. [32] have found that, in head and neck cancer, the fit of the DKI model to TW images can introduce nonnegligible bias and error in the estimation of *K* and *D* for single lesion. In particular, the median (95% confidence interval) errors in *K* and *D* were 5.1% (0.8%, 32.6%) and 1.7% (-2.5%, 5.3%), respectively. However, it is not clear how TW images affect DKI-derived indices in the estimation of *D* and *K* in the osteosarcoma. In the future, we will further investigate this potential bias. These above findings may partially explain our present results.

Our findings suggested that patients with lower post MK and higher post MD had longer PFS and OS, and this finding was also found when we evaluated the effect of these two combined parameters on survival, with highly increased risk of OS or PFS for patients in group 2 or group 3 compared with those in group 1, respectively. Increased MK indicates a higher degree of complexity of the microstructure within the tumor, which can represent tumors with higher cellular pleomorphism and more microvascular proliferation [19]. However, reduced MD reflects a higher degree of proliferation and cellularity. In good responders, reduced post MK and elevated post MD may result from alterations in cell and intracellular membrane integrity and permeability to water, cell death, or a decrease in cellularity after chemotherapy, which change the degree of restricted diffusion and heterogeneity of tumor tissue [11]. Prior studies confirmed that ^18^F–FDG PET could accurately reflect the response of osteosarcoma to chemotherapy and outcome [3, 10]. Costelloe et al. suggested that survival was associated with SUV_max_ after chemotherapy [3]. Compared to PET, DKI not only has a higher resolution but also is free from ionizing radiation, which may be well suited for dynamically evaluating osteosarcoma.

Previous studies showed that the response to chemotherapy influences on survival [33]. In our study, a weak association was observed between post MD and CR MD and OS and between post MD and PFS among the patients with PRs, while no any significant associations were found between DKI and survival among patients with GRs. Such very preliminary findings may help generate some novel hypotheses for further investigation in future larger studies.

Several potential limitations were also present in this study. Firstly, the single-center, retrospective design and small sample size may result in biased conclusions. Further validation in larger sample sizes is needed. Secondly, DKI data acquisition requires a long time, which causes patent discomfort and increases the risk of motion artifacts. Third, the design including the *b*_0_ value for DKI may increase the intravoxel incoherent motion effects. Fourth, DKI was performed by using only 3 orthogonal diffusion weighting directions. This straightforward approach does not allow to estimate the diffusion and kurtosis tensors, which are needed to adequately characterize diffusion processes and obtain rotational invariant estimates of *D* and *K*. Fifth, the use of trace-weighted images can introduce both bias and error in the estimation of DKI-derived indices of *K* and *D*. Lastly, the time course of change in DKI parameters during chemotherapy needs further exploration in the future.

In summary, DKI is a promising prognostic tool for OS and PFS in patients with osteosarcoma. Post MD, post MK, and CR MD might serve as potential invasive surrogate prognostic markers and could contribute to regimen management of individualized treatment for better survival and improved quality of life for this rare disease.

## Figures and Tables

**Table 1:** Clinical characteristics of the study patients.

Variable	*n* (%)
Age, mean (range)	17.5 (7-34)
Sex	
Male	21 (70.0)
Female	9 (30.0)
Tumor location	
Distal femur	21 (70.0)
Others	9 (30.0)
AJCC stage	
IIA	4 (13.3)
IIB	22 (73.4)
III	4 (13.3)
Surgical approach	
Limb salvage	28 (93.3)
Amputation	2 (6.7)
Pathological subtype	
Osteoblastic	27 (90.0)
Others	3 (7.0)
Huvos grade	
I and II	17 (56.7)
III and IV	13 (43.3)
Progression rate	
Free	15 (50.0)
Positive	15 (50.0)

AJCC: American Joint Committee on Cancer.

**Table 2:** DKI parameters of patients with osteosarcoma before and after chemotherapy.

Variable	Before chemotherapy	After chemotherapy	Change rate (%)
All patients (*n* = 30)			
MK	0.92 ± 0.19	0.77 ± 0.18	−12.9 ± 21.8
MD (×10^−3^ mm^2^/s)	1.29 ± 0.21	1.62 ± 0.43	26.5 ± 34.5
Poor responders (*n* = 17)			
MK	0.91 ± 0.14	0.84 ± 0.19	−7.7 ± 17.3
MD (×10^−3^ mm^2^/s)	1.29 ± 0.21	1.39 ± 0.39	7.02 ± 25.16
Good responders (*n* = 13)			
MK	0.92 ± 0.24	0.69 ± 0.14	−19.7 ± 25.8
MD (×10^−3^ mm^2^/s)	1.29 ± 0.22	1.92 ± 0.27	52.0 ± 28.1

**Table 3:** Association of DKI with the OS and PFS of patients with osteosarcoma (*n* = 30).

Variable	OS			PFS		
	Overall death/total	Log-rank	aHR (95% CI)	Pro/total	Log-rank	aHR (95% CI)
Tumor response		0.009^∗^			0.015^∗^	
GR	1/13		1.0	3/13		1.0
PR	9/17		9.4 (1.2-75)	12/17		4.2 (1.2-15)
Pre MK		0.49			0.54	
≥0.94	4/10		1.0	6/10		1.0
<0.94	6/20		0.6 (0.2-2.3)	9/20		0.6 (0.1-8.8)
Pre MD		0.83			0.63	
≥1.32	8/25		1.0	12/25		1.0
<1.32	2/5		1.1 (0.1-18.2)	3/5		1.3 (0.4-4.8)
Post MK		0.03^∗^			0.02^∗^	
≥0.80	5/9		1.0	7/9		1.0
<0.80	5/21		0.3 (0.1-0.9)	8/21		0.3 (0.1-0.8)
Post MD		0.004^∗^			0.01^∗^	
≥1.66	3/18		1.0	6/18		1.0
<1.66	7/12		5.8 (1.5-23.1)	9/12		3.5 (1.2-10.1)
CR MK (%)		0.14			0.17	
≥-16.89	5/11		1.0	7/11		1.0
<-16.89	5/19		0.4 (01-1.4)	8/19		0.5 (0.2-1.3)
CR MD (%)		0.001^∗^			0.04^∗^	
≥13.53	2/17		1.0	6/17		1.0
<13.53	8/13		8.6 (1.8-41.8)	9/13		2.9 (1.0-8.2)
Combined effect		0.068			0.052	
Group 1	3/16		1.0	5/16		1.0
Group 2	4/7		5.2 (1.1-24.2)	5/7		3.4 (0.9-11.9)
Group 3	3/7		3.0 (0.6-15.0)	5/7		3.6 (1.1-12.6)

GR: good responder; PR: poor responder; pre MK: mean kurtosis before neoadjuvant chemotherapy; pre MD: mean diffusivity before neoadjuvant chemotherapy; post MK: mean kurtosis after neoadjuvant chemotherapy; post MD: mean kurtosis after neoadjuvant chemotherapy; CR MK: change rate in mean kurtosis; CR MD: change rate in mean kurtosis in mean diffusivity; aHR: adjusted for age, sex, location, stage, pathological subtype, and treatment; HR: hazard ratio; CI: confidence interval. ^∗^*P* < 0.05. Group 1: patients with post MK < 0.80 and post MD ≥ 1.66. Group 2: patients with post MK ≥ 0.80 and post MD < 1.66. Group 3: patients with other parameters. MD is given in mm^2^/s × 10^−3^.

**Table 4:** Association of DKI with the OS and PFS of patients with osteosarcoma stratified by histological response.

Variable	OS			PFS		
	Overall death/total	Log-rank	aHR (95% CI)	Pro/total	Log-rank	aHR (95% CI)
PRs (*n* = 17)						
Pre MK		0.78			0.92	
≥0.94	4/8		1.0	6/8		1.0
<0.94	5/9		0.8 (0.2-3.3)	6/9		0.9 (0.3-2.9)
Pre MD		0.24			0.12	
≥1.32	7/15		1.0	10/15		1.0
<1.32	2/2		2.5 (0.5-12.3)	2/2		3.4 (0.6-18.9)
Post MK		0.12			0.47	
≥0.80	5/8		1.0	6/8		1.0
<0.80	4/9		0.3 (0.1-1.4)	6/9		0.6 (0.2-2.0)
Post MD		0.08			0.07	
≥1.66	2/6		1.0	3/6		1.0
<1.66	7/11		3.7 (0.7-18.7)	9/11		3.1 (0.8-11.7)
CR MK (%)		0.37			0.94	
≥-16.89	5/9		1.0	6/9		1.0
<-16.89	4/8		0.5 (0.1-2.1)	6/8		0.9 (0.3-3.0)
CR MD (%)		0.04^∗^			0.26	
≥13.53	1/5		1.0	3/5		1.0
<13.53	8/12		6.8 (0.8-58.0)	9/12		2.1 (0.5-7.8)
GRs (*n* = 13)						
Pre MK		0.67			0.43	
≥0.94	0/2		NA	0/2		NA
<0.94	1/11			3/11		
Pre MD		0.58			0.73	
≥1.32	1/10		NA	2/10		1.0
<1.32	0/3			1/3		1.5 (0.1-16.4)
Post MK		0.73			0.11	
≥0.80	0/1		NA	1/1		1.0
<0.80	1/12			2/12		0.2 (0.1-2.0)
Post MD		0.73			0.60	
≥1.66	1/12		NA	3/12		NA
<1.66	0/1			0/1		
CR MK (%)		0.67			0.44	
≥-16.89	0/2		NA	1/2		1.0
<-16.89	1/11			2/11		0.4 (0.1-4.4)
CR MD (%)		0.73			0.60	
≥13.53	1/12		NA	3/12		NA
<13.53	0/1			0/1		

aHR: adjusted for age, sex, location, stage, pathological subtype, and treatment. MD is given in mm^2^/s × 10^−3^.

## Data Availability

The data used to support the finding of this study are available form corresponding authors upon request.

## Abbreviations

DKI: Diffusion kurtosis imaging
PFS: Progression-free survival
OS: Overall survival
GRs: Good responders
PRs: Poor responders
MK: Mean kurtosis
MD: Mean diffusivity
CR MD: Change rate in mean diffusivity before and after chemotherapy
CR MK: Change rate in mean kurtosis before and after chemotherapy
DWI: Diffusion-weighted imaging
ADC: Apparent diffusion coefficient
AJCC: American Joint Committee on Cancer.
